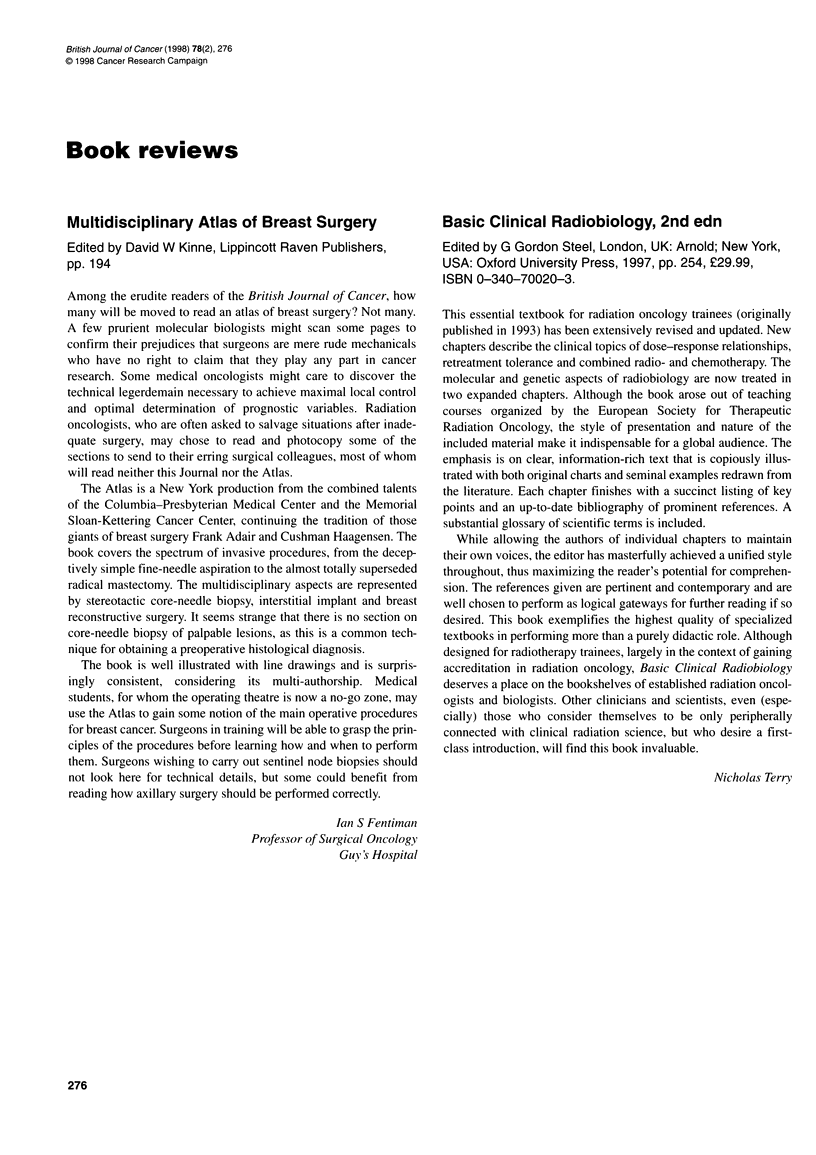# Basic Clinical Radiobiology, 2nd edn

**Published:** 1998-07

**Authors:** Nicholas Terry


### Basic Clinical Radiobiology, 2nd edn

Edited by G Gordon Steel, London, UK: Arnold; New York, USA: Oxford University Press, 1997, pp. 254, £29.99, ISBN 0-340-70020-3.

This essential textbook for radiation oncology trainees (originally published in 1993) has been extensively revised and updated. New chapters describe the clinical topics of dose–response relationships, retreatment tolerance and combined radio- and chemotherapy. The molecular and genetic aspects of radiobiology are now treated in two expanded chapters. Although the book arose out of teaching courses organized by the European Society for Therapeutic Radiation Oncology, the style of presentation and nature of the included material make it indispensable for a global audience. The emphasis is on clear, information-rich text that is copiously illustrated with both original charts and seminal examples redrawn from the literature. Each chapter finishes with a succinct listing of key points and an up-to-date bibliography of prominent references. A substantial glossary of scientific terms is included.

While allowing the authors of individual chapters to maintain their own voices, the editor has masterfully achieved a unified style throughout, thus maximizing the reader's potential for comprehension. The references given are pertinent and contemporary and are well chosen to perform as logical gateways for further reading if so desired. This book exemplifies the highest quality of specialized textbooks in performing more than a purely didactic role. Although designed for radiotherapy trainees, largely in the context of gaining accreditation in radiation oncology, *Basic Clinical Radiobiology* deserves a place on the bookshelves of established radiation oncologists and biologists. Other clinicians and scientists, even (especially) those who consider themselves to be only peripherally connected with clinical radiation science, but who desire a first-class introduction, will find this book invaluable.

*Nicholas Terry*